# PHYSICAL ACTIVITY AND SEDENTARY TIME ASSOCIATIONS WITH NON-FRACTURE FALL INJURY (NFFI) AND FRACTURE

**DOI:** 10.1093/geroni/igac059.3095

**Published:** 2022-12-20

**Authors:** Lily Bessette, Andrea LaCroix, Benjamin Schumacher, Michael LaMonte, John Schousboe, Kristine Ensrud, Jane A Cauley, Elsa Strotmeyer

**Affiliations:** University of Pittsburgh, Pittsburgh, Pennsylvania, United States; University of California, San Diego, La Jolla, California, United States; University of Pittsburgh, Pittsburgh, Pennsylvania, United States; University at Buffalo - SUNY, BUFFALO, New York, United States; HealthPartners, Inc, Park Nicollet Clinic, Minnesota, United States; University of Minnesota Medical School and Minneapolis VA Health Care System, Minneapolis, Minnesota, United States; School of Public Health, University of Pittsburgh, Pittsburgh, Pennsylvania, United States; School of Public Health, University of Pittsburgh, Pittsburgh, Pennsylvania, United States

## Abstract

Understanding fall injury risk factors and circumstances may lead to better prevention. However, many fall injury studies focus on fractures from hospitalizations or emergency events, rather than non-fracture fall injuries (NFFI), which comprise >50% of fall injuries in older adults. We hypothesized that risk factors were differently associated with NFFI vs. fracture fall injuries in a community-based cohort of ambulatory women with fall injuries in the Objective Physical Activity and Cardiovascular Disease Health in Older Women (OPACH) ancillary of the Women’s Health Initiative Long Life Study. Women with daily fall calendars over 1-year follow-up completed telephone interviews regarding fall injury circumstances (Nf662; mean 79.6 + 6.7 years; 73.3% White). Risk factors and fall circumstances were assessed with first reported fall injury (NFFI vs. fracture) using univariate and multivariate logistic regression. Participants with NFFI vs. fracture were more likely to be non-white, less likely to seek clinical treatment or need help up from fall (all p < 0.05). Adjusting for age, race, and BMI, NFFI vs. fracture were more likely to report >=6 hours sitting (OR=1.72, 95% CI=1.07–2.73) and less likely to report weekly moderate exercise (OR=0.61, 95% CI=0.38–0.96), though total accelerometer-measured sedentary time and physical activity (PA) were not significant. Self-reported PA level at the time of fall (OR=1.31, 95% CI=0.82–2.09) and walking outside for >10 minutes >= 1/week (OR=1.24, 95% CI=0.77–1.98) were not different for NFFI vs. fractures. Older women with NFFI vs. fracture had more sedentary time and less moderate exercise, which may have implications for fall injury severity.

